# Mechanical properties, and *in vitro* biocompatibility assessment of biomimetic dual layered keratin/ hydroxyapatite scaffolds

**DOI:** 10.3389/fbioe.2023.1304147

**Published:** 2023-12-15

**Authors:** Sandleen Feroz, Nawshad Muhammad, Riaz Ullah, Umar Nishan, Peter Cathro, George Dias

**Affiliations:** ^1^ School of Dentistry, The University of Queensland, Brisbane, QLD, Australia; ^2^ Department of Dental Materials, Institute of Basic Medical Sciences, Khyber Medical University, Peshawar, Pakistan; ^3^ Medicinal Aromatic and Poisonous Plants Research Center, College of Pharmacy, King Saud University, Riyadh, Saudi Arabia; ^4^ Department of Chemistry, Kohat University of Science and Technology (KUST), Kohat, Khyber Pakhtunkhwa, Pakistan; ^5^ Department of Oral Rehabilitation, University of Otago School of Dentistry, Dunedin, New Zealand; ^6^ Department of Anatomy, University of Otago, Dunedin, New Zealand

**Keywords:** alveolar bone, biomimetic, keratin, bone tissue engineering, dental implants

## Abstract

A novel biomimetic dual layered keratin/hydroxyapatite (keratin/HA) scaffold was designed using iterative freeze-drying technique. The prepared scaffolds were studied using several analytical techniques to better understand the biological, structural, and mechanical properties. The developed multilayered, interconnected, porous keratin scaffold with different hydroxyapatite (HA) content in the outer and inner layer, mimics the inherent gradient structure of alveolar bone. SEM studies showed an interconnected porous architecture of the prepared scaffolds with seamless integration between the upper and lower layers. The incorporation of HA improved the mechanical properties keratin/HA scaffolds. The keratin/HA scaffolds exhibited superior mechanical properties in terms of Young’s modulus and compressive strength in comparison to pure keratin scaffolds. The biocompatibility studies suggested that both keratin and keratin/HA scaffolds were cyto-compatible, in terms of cell proliferation. Furthermore, it showed that both the tested materials can served as an ideal substrate for the differentiation of Saos-2 cells, leading to mineralization of the extracellular matrix. In summary, ionic liquid based green technique was employed for keratin extraction to fabricate keratin/HA scaffolds and our detailed *in vitro* investigations suggest the great potential for these composite scaffolds for bone tissue engineering in future.

## 1 Introduction

Bone grafting procedures to treat the irreversible resorption of alveolar bone are widely used techniques to improve the success and durability of dental implants clinically. The recent advancements in the field of implantology have led to a significant increase in the use of bone grafting to repair craniofacial defects. Globally, the annual placement of bone grafts is greater than two million, which is predicted to show a further increase of about 13% in the coming years (Zhao et al., 2021). The dental bone grafts market has an estimated worth of approximately US $493 million and is now predicted to further increase to approximately US $931 million by 2025 (Zhao et al., 2021). Despite the extensive applications of bone grafting procedures globally, there are certain drawbacks associated with the use of grafting materials. Allografts and autografts are commonly used to treat bony defects, but they lack certain required properties to serve as an ideal bone grafting material such as high immunogenicity, high cost, donor site morbidity, procedure-related complexities and concerns regarding disease transmission (Feroz and Dias, 2021a; Feroz et al., 2023). Therefore, there is a dire demand to develop and commercialize advanced materials for bone grafting procedures to surpass the demand for autografts or allografts to treat bony defects with bone graft substitutes.

Alveolar bone has the hierarchical organization of an outer layer of dense cortical bone surrounding marrow spaces and an inner layer of trabecular bone forming a honeycomb-like structure (Yu et al., 2021). These two layers not only differ in their morphology but also show differences in porosity and rate of metabolic activities. Trabecular bone is highly porous (40%–95%) and more metabolically active than the cortical layer of bone. Overall, the density of bone increases from inside out (Ramalingam et al., 2020). Some recent advancements in the tissue engineering field have opened many new perspectives for research, but designing a novel scaffold that closely mimics the gradient structure of human alveolar bone is still a great challenge to achieve the seamless integration of multiple layers (Xu et al., 2011; Zafar et al., 2015; Ramalingam et al., 2020; Ratnayake et al., 2020; He et al., 2021). Levingstone *et al.* fabricated a layered composite structure that failed to fully achieve the biomimetic function (Levingstone et al., 2014). The poor infiltration of cells through the layers, due to the lack of a seamless integration of multiple layers, led to the limited clinical success of these scaffolds. (Levingstone et al., 2014).

Keratin is a natural polymer that possessed excellent biocompatibility, mechanical durability, and intrinsic biological activity (Feroz et al., 2020). This autogenous protein has been extensively studied in the field of tissue engineering and can be extracted from various natural sources such as hair, wool, horns, and feathers (Feroz and Dias, 2021a). Among these sources, wool-derived keratin offers several advantages as there is no risk of pathogen transfer, no cultural/religious constraints which are mostly associated with the use of materials from bovine/porcine origin (Goyal et al., 2013). Despite having all these desirable properties, the fragile nature of keratin-based biomaterials is a major challenge for tissue engineers to address for the successful utilization of these biopolymers for bone tissue engineering (Feroz and Dias, 2021a). Recently, our group proposed a novel approach to design a keratin/HA scaffold using hydroxypropyl methyl cellulose (HPMC) as a cross-linking agent (Feroz and Dias, 2021a). Previously, it has been used as a gelation agent in the preparation of chitosan/HA scaffold, as a plasticizer to fill bony defects, in bone cement to further enhance their strength, as a cross-linking agent to develop grafts for bone regeneration (Virto et al., 2003; Urban et al., 2004; Zhang et al., 2016; Zeeshan et al., 2018).

Similarly, hydroxyapatite (HA) is a calcium phosphate based ceramic material possessing excellent biocompatibility, osteoconductivity, and strong affinity to natural polymers. Due to these, it has been studied extensively as a grafting material to treat bony defects in several studies (Kattimani et al., 2016; Feroz and Khan, 2020; Feroz and Dias, 2021a). However, some of the major limitations associated with the use of hydroxyapatite as a bone substitute in load bearing areas are its low mechanical strength and its inherent brittleness (Fan et al., 2018). Mixing of hydroxyapatite and keratin in different ratios to closely emulate the natural bone serves as a successful approach in designing a porous 3—dimensional scaffold with improved mechanical strength for *in vivo* implantation for bone regeneration (Dias et al., 2017; Fan et al., 2018).

Unlike collagen, there is still dearth of information in the successful application of the keratin derived biomimetic scaffold for bone tissue engineering (Dias et al., 2010; Feroz and Dias, 2021a). Our present study is an ongoing effort to further explore the use of an ionic liquid based non-toxic, green processing technique for keratin extraction to manufacture keratin/HA scaffolds containing dual gradient pore size and hydroxyapatite content. Thus, the novelty of the current research study is the extraction of keratin using ionic liquid based green processing technique for its application for hard tissue regeneration (Feroz et al., 2022). To the best of our knowledge, there have been no studies reported so far with keratin extracted by ionic liquid-based method to serve as major organic component for the fabrication of layered scaffold by using an iterative freeze-drying technique.

## 2 Experimental

### 2.1 Materials and methods

Wool fibres were provided by wool services international (New Zealand). Ionic liquid, nano hydroxyapatite powder (<200 nm particle size), MTS cell proliferation assay kit, phosphate buffered saline (PBS) tablets, dexamethasone, acetic acid (glacial, ˃ 99.7%), beta–glycerophosphate (β–GP), L–ascorbic acid-2-phosphate were supplied by Sigma Aldrich (Auckland, NZ). Other chemicals including trypsin, streptomycin and penicillin were purchased from Thermo Fisher scientific-AU.

### 2.2 Extraction process of keratin using ionic liquids

Ionic liquid assisted probe sonication method was used for keratin extracted from cleaned wool fibres (Feroz et al., 2022). Briefly, wool fibres were cleaned and defatted. The small pieces of fibres about 5–10 mm were immersed in 10 mL of ionic liquid at room temperature. A 20% (w/v) concentration of wool fibre was sonicated for about 25 min to achieve the maximum percentage dissolution of wool in ionic liquid (Feroz et al., 2022). To lower the viscosity of ionic liquid and wool mixture, 3 volumes of dimethyl sulfoxide (DMSO) was added to ensure complete dissolution. The mixture was centrifuged at 4000 rpm for 15 min. The residue collected was washed, dried, and weighed to determine the percentage dissolution. For regeneration of dissolved keratin, 30 mL of deionized water was added to the wool/ionic liquid solution followed by high-speed centrifugation (12000 rpm) for 15 min. The keratin solution was dialyzed and freeze-dried overnight to obtain pure keratin powder for further testing.

### 2.3 Keratin/HA bi-layered scaffolds preparation

Several preliminary experiments were conducted to investigate in detail the effect of varying concentrations of hydroxyapatite, HPMC, pre-freezing temperatures, and duration of freeze drying on the final prepared scaffold. Before optimizing the most effective method to fabricate a layered scaffold, individual layers were freeze dried and prepared separately to finalize the composition and design of the dual-layered biomimetic scaffold. Two different concentrations, each having different weight% of keratin/HA suspensions were prepared to fabricate dual gradient scaffolds having a higher concentration of hydroxyapatite (60%) in the upper and lesser concentration of hydroxyapatite (40%) in the lower layer.

Briefly, to prepare the lower layer, HPMC (250 mg) was added into the mixture containing keratin and was mechanically stirred for 6 h at room temperature. In a separate beaker, nano hydroxyapatite powder (400 mg) was added to deionized water and the suspension was sonicated at 30% amplitude for 15 min followed by addition to the crosslinked keratin mixture. Finally, the mixture was mechanically stirred overnight at room temperature to obtain complete homogenization. The upper layer suspension was prepared by the same procedure except for the concentration of hydroxyapatite which was increased up to 600 mg. The final composition (in terms of weight %) of upper layer of porous dual gradient keratin/HA/HPMC scaffolds was keratin (100%), HA (60%), HPMC (25%), whereas the composition of lower layer was keratin (100%), HA (40%) and HPMC (25%) respectively.

**FIGURE 1:**
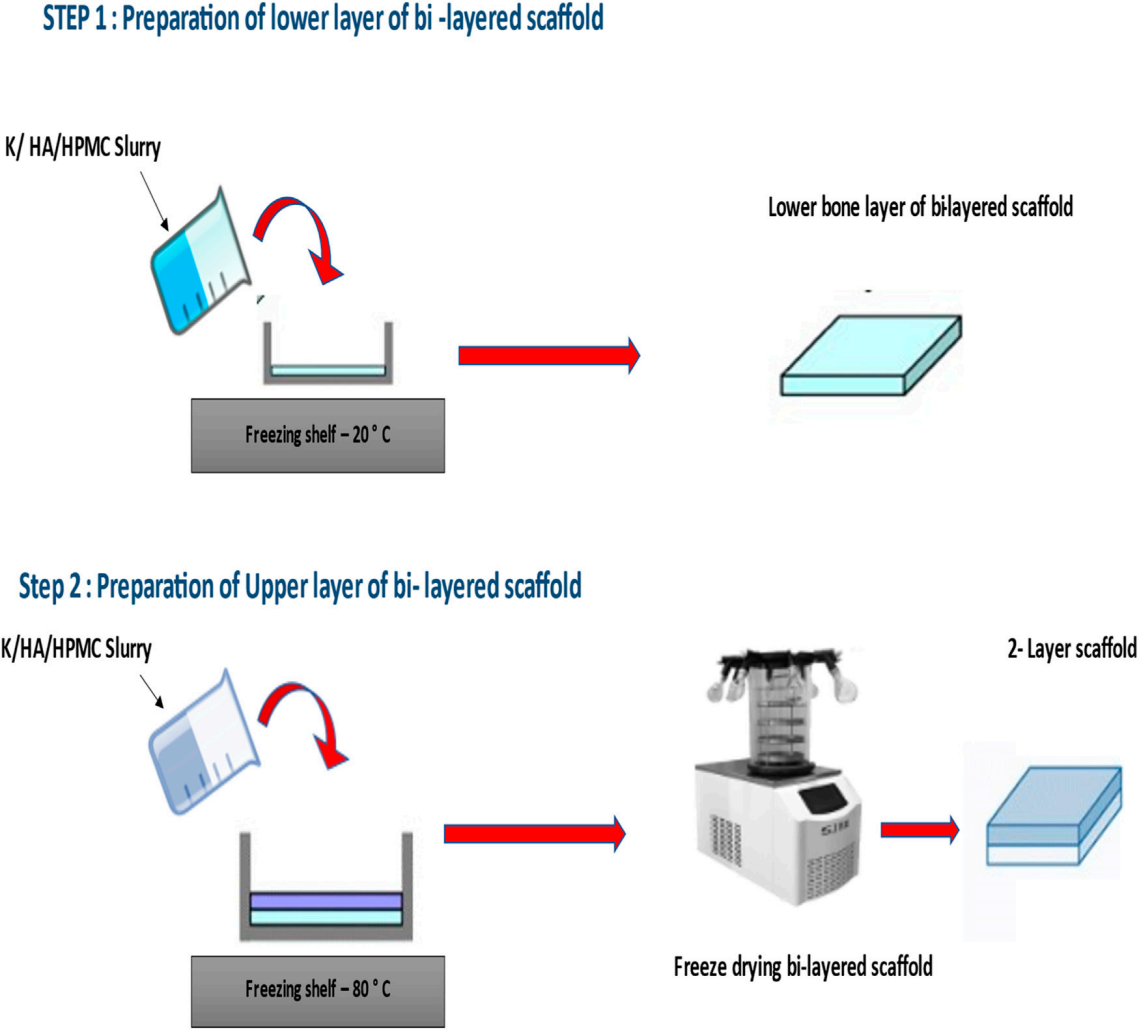
Preparation of Keratin/hydroxyapatite bi-layered scaffold using HPMC as a cross-linking agent.

The composite dual gradient scaffolds were prepared by using the “layer by layer” freeze drying technique at different pre-freezing temperatures for each layer. This simple technique mainly involves the freezing of the lower layer at −20°C temperature for 48 h. After freezing the layer was taken out from −20°C temperature for 3 min s followed by pipetting the upper layer. Finally, this multilayer scaffold was placed at −80°C for 48 h. Then, the scaffolds were freeze-dried at a final freezing temperature of −50°C for 18 h to obtain 3-dimensional dual gradient scaffolds. For control group pure keratin scaffolds were prepared. All the prepared samples were placed in a sterile environment at room temperature before testing. The final optimized iterative freeze-drying method to prepare keratin/HA scaffolds is presented schematically in Figure 1.

### 2.4 Characterization

The morphological, mechanical, and *in vitro* biological characterizations of the prepared scaffolds were conducted using various analytical techniques. The other detailed methods used for the physical and chemical characterization is mentioned in the supplementary/supporting data section below.

#### 2.4.1 Scanning electron microscopy (SEM)

The surface morphology and microstructure of the layered scaffolds was studied using a scanning electron microscope equipped with an EDX analyser (JOEL 2300; Oxford, UK). To better understand the pore size and surface morphology, both upper and lower layers of the dual gradient scaffold were visualized under different magnifications. Additionally, transverse sections of the bilayered samples were also analysed to better understand the structural continuity at the interfaces. All prepared samples were gold coated (80 A°) and studied under SEM at an accelerating voltage of 20 kV.

#### 2.4.2 Mechanical properties

Instron universal testing machine was used to better understand the mechanical properties of the composite scaffolds (thickness 9mm, diameter 18 mm). For compression measurements, all the prepared samples were placed between the platens, and a 1 kN load was applied in a downward direction at a crosshead speed of 0.5 mm/min. The exponent software (version v6.1.5.0) was used to measure the compression stress-strain curve for Young’s modulus determination. To calculate the Young’s modulus, the most linear part of the stress-strain curve was considered as the presence of any surface irregularities could produce a non-linear graphical presentation.

#### 2.4.3 Biocompatibility assessment

The Saos-2 (Human Osteosarcoma Cells ATCC HTB-85) of four to six passages were used for biocompatibility assessment in terms of cellular proliferation, adhesion, and differentiation. Minimum essential media alpha (MEM-α; Gibco™/Life Technologies, Auckland, NZ) supplemented with 10% fetal bovine serum (FBS; Sigma-Aldrich, NZ) and 1% antibiotics (Thermo Fisher Scientific, NZ) was used to cultured cells in a humidified atmosphere (5% CO₂) at 37°C. All these experiments were performed in triplicates.

#### 2.4.4 Cell proliferation assay

The standard MTS assay was carried out to measure the cellular proliferation on tested scaffolds at a seeding density of 6 × 10³ cells/scaffolds (Rodan et al., 1987). This calorimetric technique measured the metabolic level of live cells by reducing the tetrazolium component of the MTS assay. Thus, the colour change was observed due to the reduction of tetrazolium dye to formazan by cellular enzymes. A spectrophotometer (Labtech LT 4500 microplate reader) was used to determine the cellular proliferation by measuring absorbance at the wavelength of 490 nm after 24, 48, and 72 h.

#### 2.4.5 Cell attachment and morphology

The adhesion and morphology of the seeded cells on to the tested scaffolds were visualised using SEM after incubation time of 24, 48 and 72 h. These scaffolds were fixed with 3.5% glutaraldehyde in cacodylate buffer (CB). All scaffolds were rinsed again with PBS, CB buffer, and post-fixed with osmium tetroxide (1%). Serial dilutions of ethanol solution (30%, 50%, 70%, 90% and 100%) were used for dehydration for about 10 min. The samples were sputter-coated with cold palladium coating after mounting on an aluminium stubs and SEM images were captured at an accelerating voltage of 15 kV.

#### 2.4.6 Cell differentiation

##### 2.4.6.1 Alkaline phosphatase activity

Alkaline phosphatase (ALP) activity was measured to determine the cell differentiation after being cultured for 3, 7, 14, and 21 days in differentiation media. The seeding density for differentiation studies was 20 × 10³ cells/scaffold. The fresh batch of differentiation media was prepared before each experiment by adding L-ascorbic acid 2 -phosphate (100 µM), dexamethasone (10 nM), β-glycerophosphate (β-GP) to the Saos-2 growth medium (S-GM). SensoLyte^®^ pNPP Alkaline Phosphatase Assay kit (Ana Spec/EGT Group, California, USA) was used for quantitative analysis of ALP activity according to the manufacturer’s instructions. The transformation of p-nitrophenyl phosphate (pNPP) into p-nitrophenol in the presence of ALP was measured by using a spectrophotometer (BioTek, VT, USA) at 410 nm. All the experiments were performed in triplicate.

##### 2.4.6.2 Alizarin red assay (ARS)

ARS staining was performed to quantify the deposition of mineralised nodules by the differentiated cells after being cultured for 21 days in osteogenic media (S-DM). In brief, after being cultured for 21 days, the cell-seeded scaffolds were washed thrice with PBS followed by fixing with 4% paraformaldehyde solution for 10 min. The cells were washed again with PBS and stained with ARS (40 mM) for 1 h at a pH of 4.2. Subsequently, all the samples were gently washed with distilled water to remove the non-specifically bound excess dye. All the samples were photographed (Canon Powershot, Japan). Furthermore, all the samples were visualised under phase-contrast microscope (EVOS M5000, Invitrogen, Thermo Fisher Scientific) to examine the ARS-stained calcium deposits in the extracellular matrix of differentiated osteoblasts. Cell mineralization was measured quantitatively to detect the deposition of calcium rich deposits by extracting alizarin red with 10% cetylpyridinium chloride (Sigma-Aldrich) (Gao et al., 2016). Thus, the bound alizarin red stain was eluted with cetylpyridinium chloride (CPC) in sodium phosphate (100 mM) and transferred to a 96-well plate to measure the solution absorbance at 562 nm using Synergy 2 Multi-Mode Microplate Reader (Biotek^®^).

##### 2.4.6.3 ELISA testing for osteocalcin detection

The osteoinductive character of the keratin and keratin/HA scaffolds was assessed to quantify the expression of specific bone marker, osteocalcin, using the Osteocalcin ELISA™ kit (Thermo Fisher Scientific). This osteocalcin Immunoassay is an enzyme-linked immunosorbent technique designed to detect the presence of human osteocalcin quantitatively. A microplate has been pre-coated by a monoclonal antibody specific for human osteocalcin. All the samples and standards were pipetted into the microwells as per the manufacturer’s instructions. Any osteocalcin present in the samples was bound by the immobilized antibody. An enzyme-linked monoclonal antibody (specific for osteocalcin) has been added. Any unbound enzyme conjugated anti-human osteocalcin was removed during a washing step. A substrate solution was added to all wells and colour development was observed. The intensity of this colour is proportional to the concentration of osteocalcin present and got bound initially. The acid was added to terminate the reaction and final absorbance was measured at 450 nm by using a Microplate reader (Synergy 2 Multi-Mode Microplate Reader, Biotek^®^, VT, USA).

## 3 Results and discussions

### 3.1 Morphological analysis of scaffold using scanning electron microscope (SEM)

**FIGURE 2:**
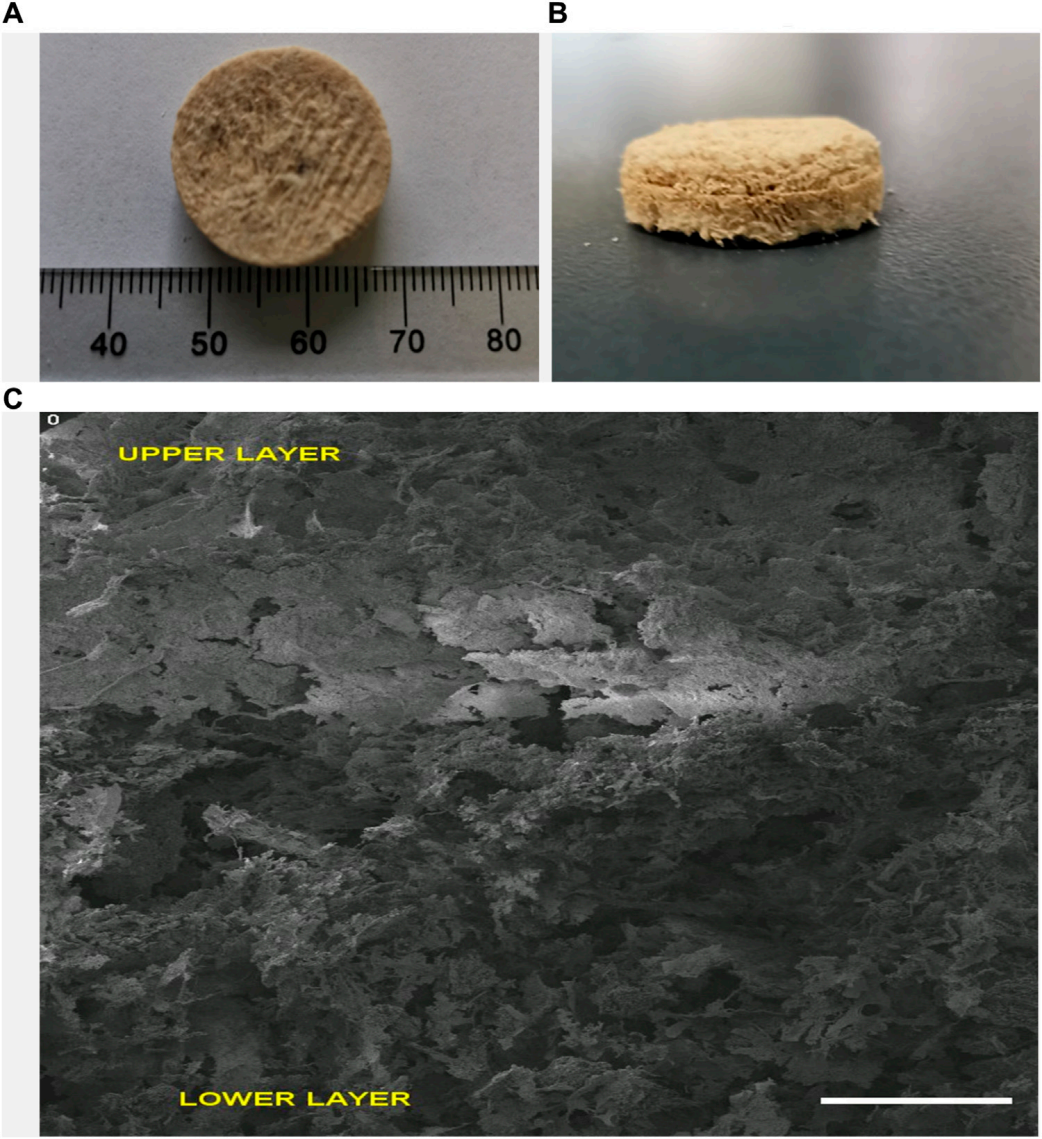
Multilayered Keratin-HA scaffold general appearance (diameter: 18 mm, height 9 mm) **(A,B)**. Vertical section of multilayered scaffold **(C)**. Scale Bar = 1 mm.

After fabrication, surface morphology and pore size evaluation of the bi-layered scaffold was done initially with the aid of scanning electron microscopy (SEM) as shown in Figure 2 and3. An interconnected porous structure was observed with seamless integration between the upper and lower layers as shown in Figure 2C. This structural continuity is vital between the upper and lower layers to promote cellular infiltration and tissue regeneration at different levels of the scaffold. This porous, 3-dimensional scaffold was designed by using an iterative freeze-drying technique to closely mimic the natural structure of alveolar bone having an outer compact and inner trabecular bone. The highly porous interconnected structure of our fabricated scaffolds could play a significant role in new bone formation as it would enhance the process of osteogenesis (Feroz et al., 2020; Feroz and Dias, 2021a).

**FIGURE 3:**
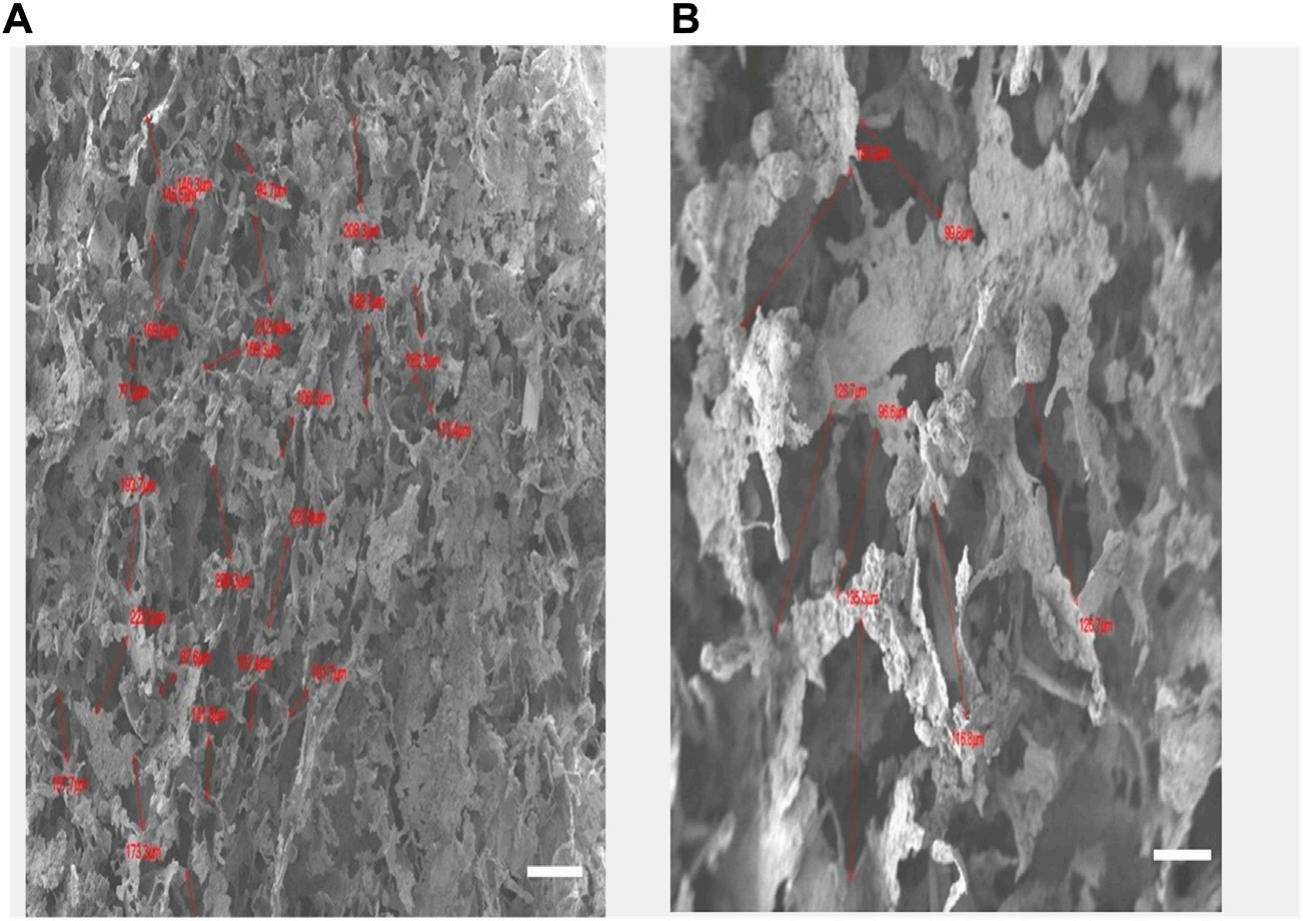
**(A)**: Keratin hydroxyapatite scaffold upper layer containing 60% of HA and the lower layer with HA content of 40% **(B)**. Scale Bars 100 µm.

Our results indicate that pore size varies considerably by changing the concentration of nano-HA and by varying the freezing temperature. The lower layer containing 40% HA and frozen at −20°C exhibited an average pore size of 136 ± 4.62 um while the upper layer containing 60% HA and frozen at −80°C showed a much denser structure with pore size decreased to an average of about 102 ± 6.92 um as shown in Figure 3 A and B. The layered-by-layered freeze-drying technique enabled the ability to control the pore size from top to bottom of the scaffold (Reys et al., 2017). Thus, as the HA content increased from the bottom to top layer, the pore size decreased accordingly. Our results are in accordance with the previous studies in which *Chen et al.* observed that the average pore size of the multilayered scaffold can be controlled by freezing at different temperatures (Chen et al., 2017).

Overall, SEM analysis demonstrates that the multi-layered keratin/HA scaffold having different pore sizes and dual concentration of HA closely emulates the gradient structure of natural alveolar bone in which there is a gradual increase in density from inside out.

### 3.2 Mechanical testing

The mechanical properties of the 3-dimensional layered scaffold are mainly governed by the inherent compressive modulus of the organic component, presence of crosslinker, and interfacial interaction of the organic and inorganic phases. Previous studies have suggested that biomechanics of scaffolds is an important factor from a clinical perspective (Feroz et al., 2020; Feroz and Dias, 2021a; Li et al., 2021). These scaffolds should provide stable fixation for efficient regeneration of new bony tissues. All factors related to the structural designing of these biomimetic scaffolds such as shape, porosity, pore diameter, seamless integration of layers, directly influence the final compressive strength (Wang et al., 2010). It was demonstrated by *Zaman et al.* (Zaman et al., 2005) that the stiffness of the matrix can change the cell movement. In this study, two substrates having the same topographic features, but different stiffness were investigated. It was found that there was enhanced elongation of endothelial cells on the soft poly dimethyl siloxane (PDMS) substrates compared to stiff SiO₂ substrates (Zaman et al., 2005). Recently our group optimized the stiffness of keratin/HA scaffolds by employing HPMC as a cross-linker for designing scaffolds for alveolar bone regeneration (Feroz and Dias, 2021a). This multi-layered scaffold is the continuity of that ongoing investigation to fabricate composite scaffolds closely emulating the natural architecture of alveolar bone.

**FIGURE 4:**
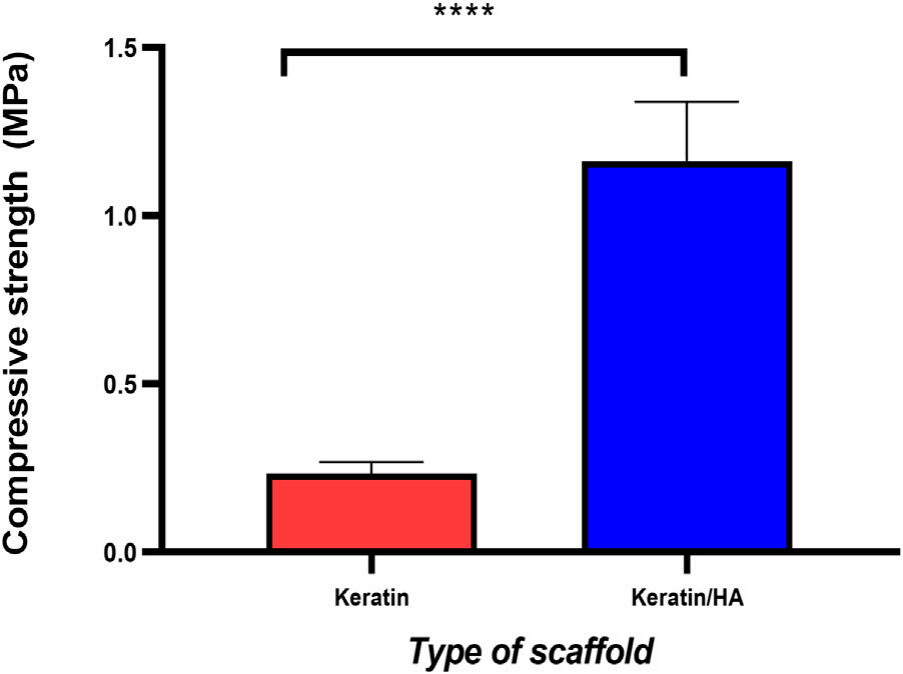
Compressive strength (MPa) of keratin and keratin/hydroxyapatite composite scaffolds (n = 10 per scaffold, Error bars represent ±SE of the mean, *t*-test, **** P ˂ 0.0001).

**FIGURE 5:**
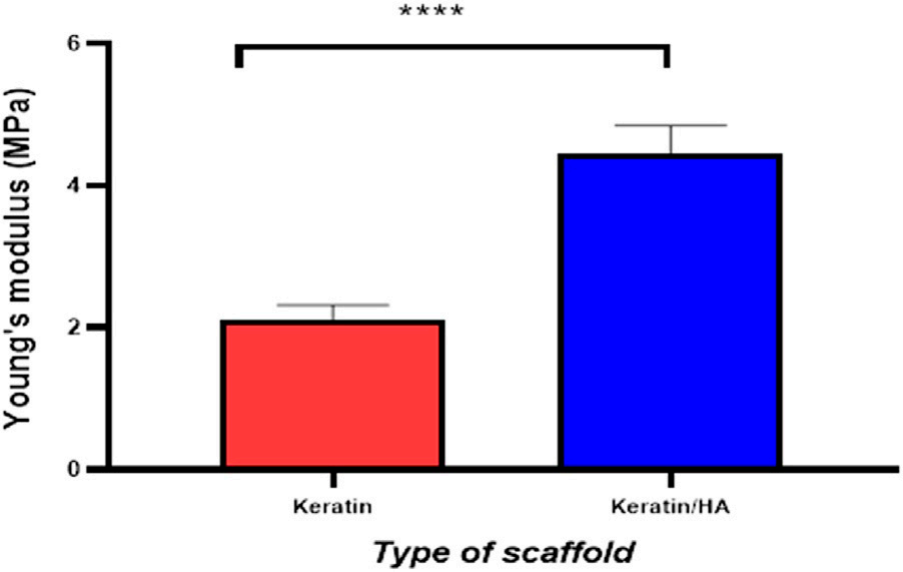
Graphical representation of the Young’s modulus of keratin and keratin/Hydroxyapatite scaffolds (n = 10, Error bars represents ± SE of the mean, *t*-test, **** P˂ 0.0001).

Figure 4 and Figure 5 represent the mechanical properties (compressive strength and young’s modulus) of keratin and keratin/HA scaffolds. The lyophilized keratin scaffolds were fragile and showed a compressive strength of 0.23 ± 0.01 MPa. However, compressive strength was significantly improved by the addition of hydroxyapatite as shown in Figure 4. The mean compressive strength value of keratin/HA was 1.16 ± 0.05 MPa. Young’s modulus of pure keratin scaffold was 2.09 ± 0.07 as illustrated in Figure 5. The Young’s modulus obtained for keratin/HA scaffolds was 4.45 ± 0.12 MPa which is significantly higher than the values reported for keratin scaffolds.

Several studies have reported that the uniform distribution of hydroxyapatite particles in the crosslinked polymeric matrix could improve the biomechanical properties of the final construct (Kane and Roeder, 2012; Bavaresco et al., 2020; Feroz and Dias, 2021a; Gurumurthy and Janorkar, 2021). In this study, the compressive strength and Young’s modulus for the keratin/HA scaffold were 1.16 MPa and 4.45 MPa respectively. These values are higher compared to previous studies (Fan et al., 2018; Feroz et al., 2020). According to the study reported by Fan *et al*, the highest compressive strength and Young’s modulus values achieved after reinforcement treatment on keratin/HA scaffolds were 0.778 MPa and 3.3 MPa (Fan et al., 2018). Similarly, Feroz *et al.* found the compressive strength for a HPMC cross-linked keratin/HA scaffold to be 0.84 MPa (Feroz and Dias, 2021a). The iterative freeze-drying technique used in this study for the fabrication of dual gradient biomimetic scaffold resulted in improved mechanical properties of the final construct. In addition to improved mechanical properties, the seamless integration of two layers would also potentially promote the cellular infiltration and regeneration of new tissue.

### 3.3 Cell proliferation

**FIGURE 6:**
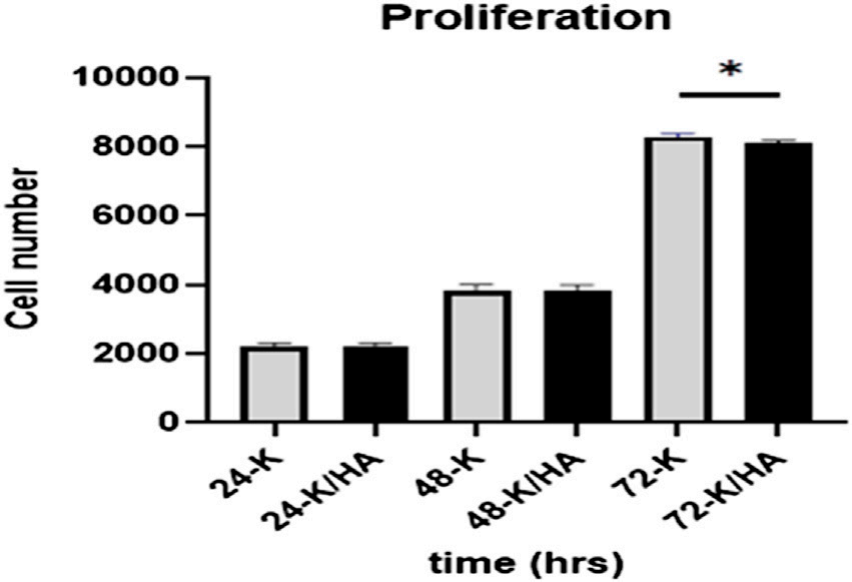
Graphical representation showing Saos-2 cell proliferation on keratin and keratin/Hydroxyapatite scaffolds at three tested time points of 24, 48 and 72 h (Error bars are presented as the standard error of the mean, n = 3, * P ˂ 0.05).

The cells proliferation was investigated using the MTS assay at 24, 48, and 72 h, respectively. At 24 and 48 h, no significant differences were observed for Saos-2 cell proliferation for keratin/HA compared to keratin scaffolds (*p* = 0.6). At 72 h, cell number observed for keratin scaffolds was significantly higher compared to keratin/HA scaffolds (*p* = 0.03) as shown in Figure 6. These results demonstrate that both the keratin and keratin/HA scaffolds support cell proliferation.

These aligned with the previous studies that have reported decreased cellular proliferation due to the osteogenic differentiation of cells by hydroxyapatite. (Eosoly et al., 2012; Chen et al., 2017; Feroz and Khan, 2020; Feroz and Dias, 2021a). Similarly, Eosoly *et al.* also observed significantly lower cell proliferation for composites with high hydroxyapatite content (Eosoly et al., 2012). We also postulate that the presence of a high concentration of hydroxyapatite in the keratin/HA composite induces the osteogenic differentiation of cells leading to a decrease in cell proliferation. It has been reported previously that hydroxyapatite significantly increases the hydrophilicity of the scaffolds which makes it more bioactive, hence an excellent template for cellular attachment and proliferation (Al-Munajjed et al., 2009; Akkouch et al., 2011; Chen et al., 2017). The next phase was to evaluate the osteoinductive properties of the prepared scaffolds as both tested scaffolds showed excellent biocompatibility by promoting cellular attachment and proliferation.

### 3.4 Evaluation of cell adhesion/morphology

Scanning electron microscope was used to evaluate the cellular morphology and adhesion on the scaffold surfaces at 72 h of incubation.

**FIGURE 7:**
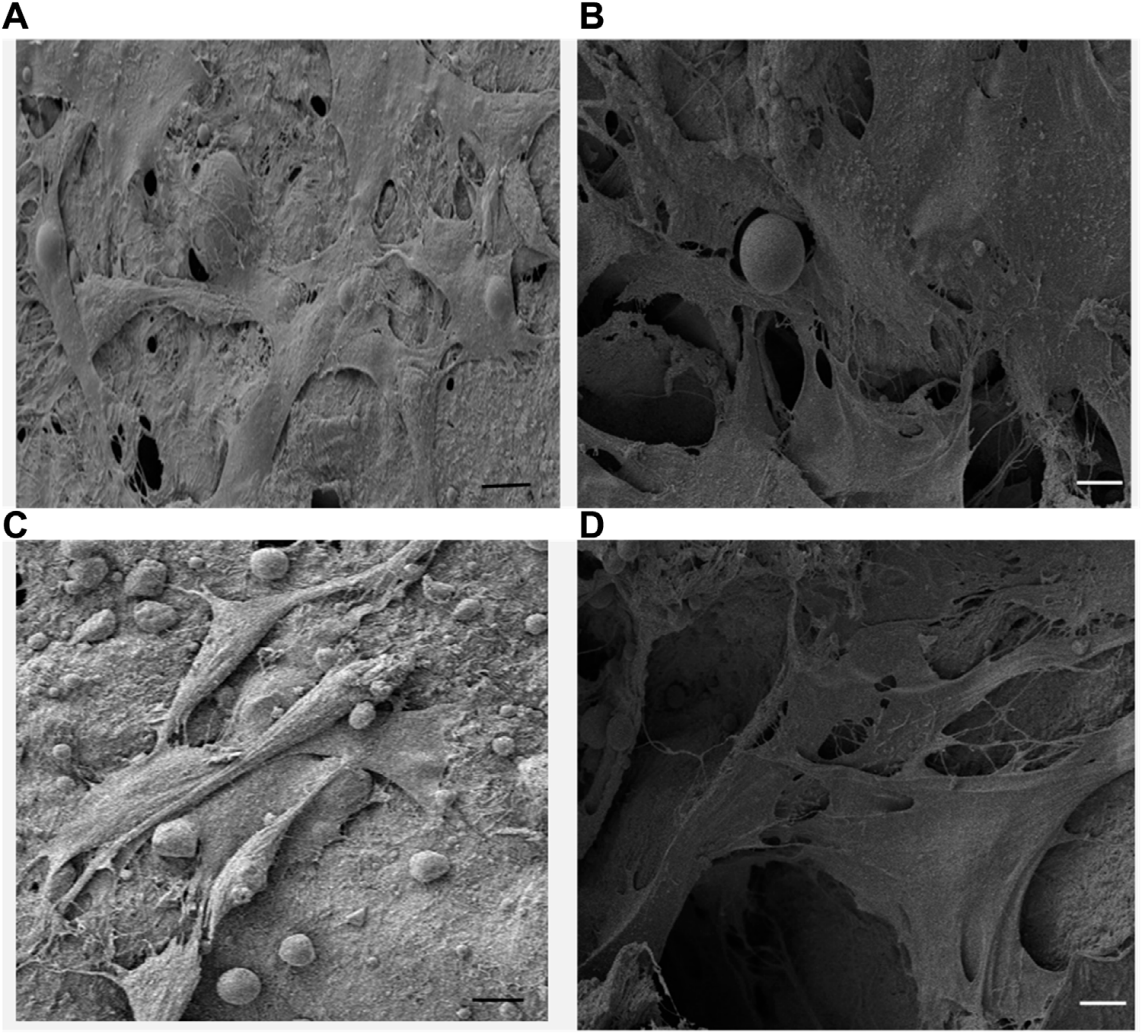
**(A,C)** SEM micrographs of Saos-2 cells on keratin scaffolds showing the cell attachment and the formation of mesh like network on the surface and within the porous structure, **(B,D)** Saos-2 cell adhesion on keratin/HA scaffolds facilitated by long cytoplasmic extensions within the porous 3-dimensional matrices. (Scale bars represents 10 µm).

Figures 7A, C shows the sheet-like morphology of the adherent cells on the surface of the scaffolds. The topographic features of the surface of the scaffold play a crucial role in cell attachment and the observed results showed strong affinity of the cells towards both tested scaffold surfaces (Gumbiner and Yamada, 1995). Thus, sheet like morphology observed under SEM involving multiple cells is the initial step towards multilayer structure formation. (Feroz and Dias, 2021a).

It was also observed that the cells formed cytoplasmic extensions, which appeared to form a network of microfilaments between the cells and the surface of the scaffolds. The cross-sectional SEM image of the cell-seeded scaffolds showed that the cells were able to adhere, migrate and proliferate through the interconnected porous structure and present cytoplasmic extensions of 30–50 µm of length (Figures 7B, D). The highly porous interconnected structure of the keratin/HA bi-layered scaffold facilitates cellular adhesion and enhances the process of new bone formation.

### 3.5 Alkaline phosphatase activity

ALP activity is an important phenotypic marker of osteoblasts which peak during the early stages of osteoblast differentiation (Igarashi et al., 2004). Initiation of calcination at the sites of nucleation accumulates more phosphate and calcium ions. (Ogata et al., 2005). During this process, ALP hydrolyses the phosphate esters and the concentration of local phosphate ions increases which further stimulates the process of extracellular matrix mineralization (Lian, 1996). In this study observational periods of 3, 7, 10, and 14 days were selected which is based on the literature investigating osteoblast differentiation (Prideaux et al., 2014; Chen et al., 2017).

**FIGURE 8:**
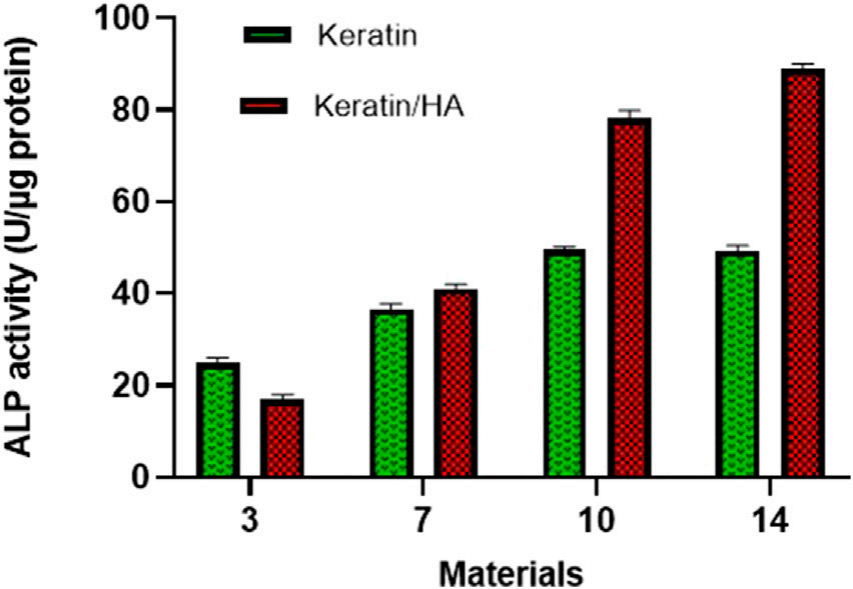
ALP activity of Saos-2 cells on keratin and keratin/Hydroxyapatite scaffolds after three predefined time points of 3,7 and 14 days (n = 3, Error bars present ± SE of the mean).

Figure 8 shows the ALP activity of the keratin and keratin/HA scaffolds on days 3, 7, 10 and 14. Keratin/HA scaffolds showed a significantly lower level of ALP activity on day 3 compared to pure keratin scaffolds (*P* ˂ 0.001). However, when ALP expression was measured for both scaffolds from day 7 to day 14, a significantly higher level of ALP activity was observed for keratin/HA scaffolds compared to pure keratin scaffolds. Furthermore, there was no statistically significant difference observed in ALP expression for keratin scaffolds on both days 10 and 14. These result shows that both keratin and keratin/HA supporting osteogenic differentiation of Saos-2 cells. Several studies have demonstrated that the composition and the porosity of the tested material play a vital role in promoting the formation of cellular clusters which then lead to the differentiation of cells (Gao, 2006; Taniguchi et al., 2016; Feroz and Dias, 2021b). It is also evident by these results that keratin/HA scaffolds possessing a stronger ability to promote the osteogenic differentiation of Saos-2 cells when compared to pure keratin scaffolds (Figure 8).

It has been reported previously that the presence of hydroxyapatite significantly improving cellular activity which has a positive influence on cell differentiation and mineralization (Chen et al., 2017; Zhao et al., 2018; Feroz and Dias, 2021a). Thus, the presence of hydroxyapatite could enhance the Saos-2 cells osteogenic differentiation. A study conducted by Chen *et al.* found that the presence of hydroxyapatite in a collagen/hydroxyapatite scaffold induces the differentiation of cells (Chen et al., 2017). These findings are consistent with current results which also manifested an elevated level of ALP activity of cells in keratin/HA scaffolds compared to the keratin scaffolds.

### 3.6 Alizarin red assay (AR-S)

The formation of calcium deposits indicates the differentiation of cells into the mineralization phase to produce a mineralized extracellular matrix (Chen et al., 2017). ALP is an early-stage marker for osteogenic differentiation of cells whereas calcium deposition is mainly associated with the late stage of osteogenic differentiation. In the current study, ARS staining was used to characterize the calcium deposition of differentiated Saos-2 cells cultured on keratin and keratin/HA scaffolds. The ARS staining was followed by extracting ARS with 10% cetylpyridinium chloride (CPC) for the quantitative assessment of calcium deposition. Based on the literature, an observational period of 14 and 21 days was selected to investigate the mineralization potential of tested biomaterials (Maharjan et al., 2021).

**FIGURE 9:**
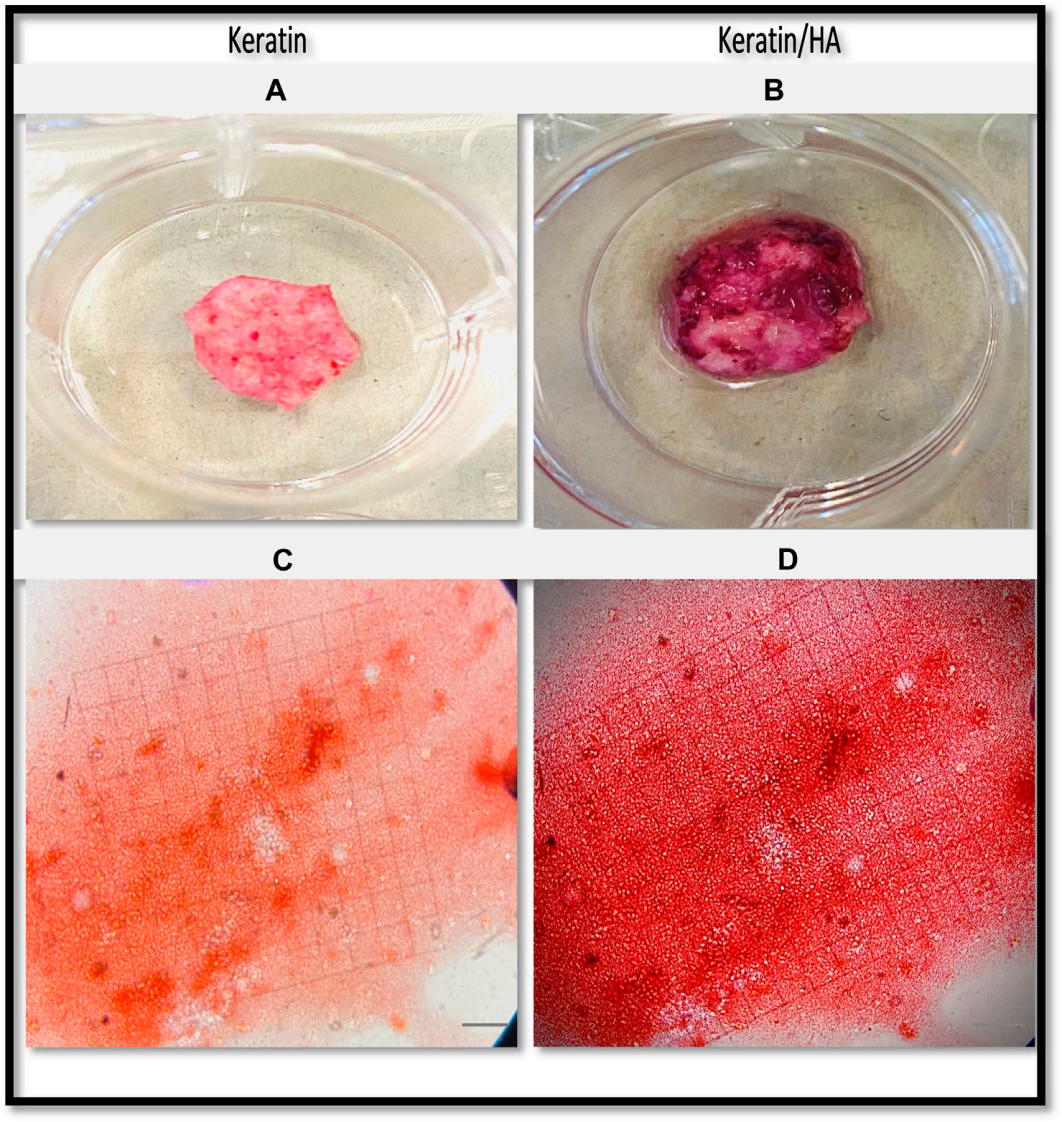
Optical images of the Alizarin Red S-stained keratin **(A)** and keratin/HA scaffolds **(B)**. Phase contrast images of Alizarin Red S-stained mineralized nodules formed by Saos-2 cells on keratin **(C)** and keratin/Hydroxyapatite scaffolds **(D)** over a culture period of 14 days. (Original magnification: ×200; Scale bars = 20 µm)*.*

**FIGURE 10:**
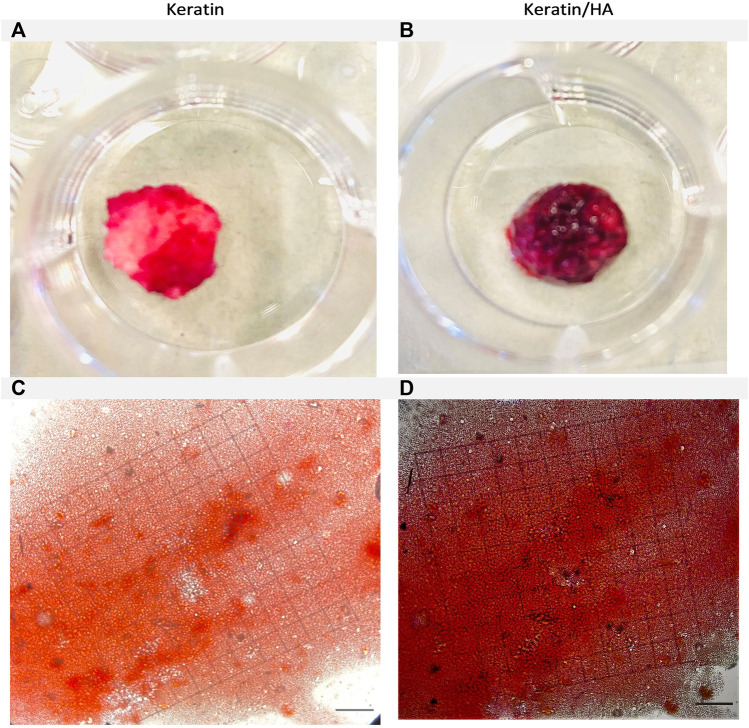
Optical images of the Alizarin Red S-stained keratin **(A)** and keratin/Hydroxyaptite scaffolds **(B)**. Phase contrast images of Alizarin Red S-stained mineralized nodules formed by Saos-2 cells on keratin **(C)** and keratin/Hydroxyapatite scaffolds **(D)** over a culture period of 21 days. (Original magnification: ×200; Scale bars = 20 µm)*.*

Figures 9A, B and Figures 10A, B shows the optical images of keratin and keratin/HA scaffolds stained with ARS dye at day 14 and 21. To further evaluate the formation of calcium-rich deposits on the ARS-stained scaffolds, the samples were viewed by phase contrast microscope as shown in Figures 9C, D and Figures 10C, D. A red colour stain was observed for all the tested samples, but keratin/HA scaffolds showed a more pronounced bright red staining than keratin scaffolds on both days 14 and 21. These results are in accordance with previous studies in which it was observed that the presence of HA accelerates the process of osteogenesis by enhancing the osteoblast differentiation (Madurantakam et al., 2009; Chen et al., 2017; Park et al., 2021).

**FIGURE 11:**
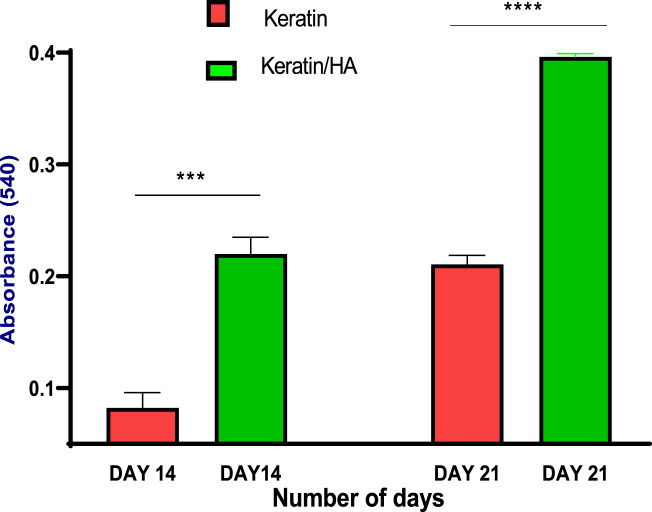
Quantitative analysis of calcium deposits by measuring the absorbance of the extracted Alizarin Red dye at day 14 and 21 (n = 3, *** *p* = 0.0003, ****P˂0.0001, error bars represent ± SE of the mean).

The quantification of calcium deposits determined by extracting ARS with CPC (10%) showed a significantly higher calcium content for keratin/HA scaffolds compared to pure keratin scaffolds on day 14 (Figure 11). A similar trend was observed on day 21, where the total calcium content was about 50% higher than that in the pure keratin scaffold. Consistent with the findings of ALP analysis, the results of qualitative and quantitative analysis of cell mineralization showed a higher concentration of calcium mineral deposition by keratin/HA scaffolds.

### 3.7 ELISA testing for osteocalcin detection

Osteocalcin or Bone Gla Protein (BGP) is the major non-collagenous protein synthesized by osteoblasts (Hauschka and Wians, 1989). It serves as an excellent late-stage biochemical marker for osteogenic maturation. Furthermore, several studies have reported an upregulated level of osteocalcin during the mineralization period (Hauschka and Wians, 1989; Stein et al., 1996).

**FIGURE 12:**
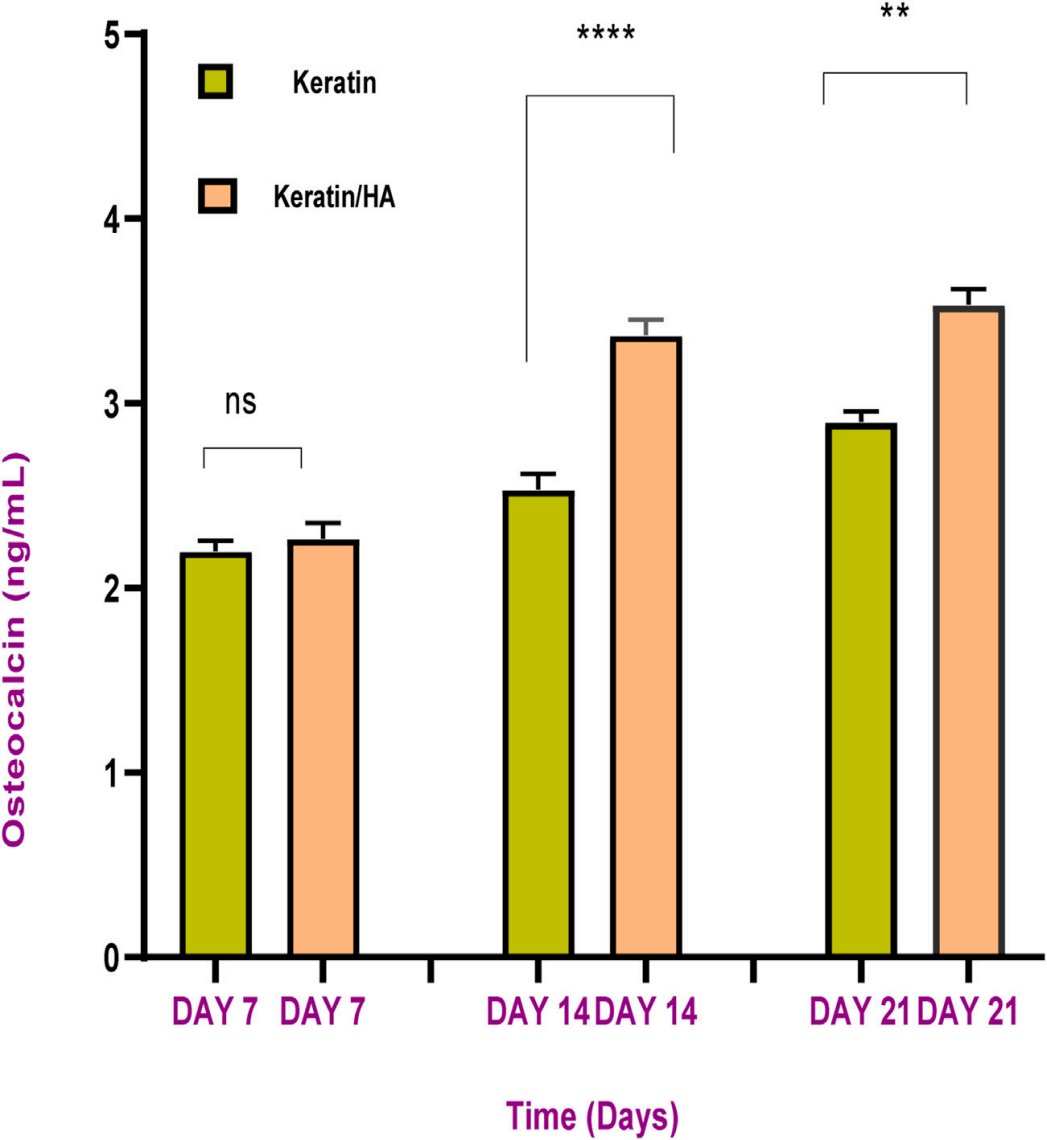
Osteocalcin content of keratin and keratin/Hydroxyapatite scaffolds at different cultured time (n = 3, error bars represent ± SE of the mean, **** P˂0.0001, ** *p* = 0.0012).

The sandwich enzyme-linked immunosorbent assay (ELISA) was conducted to measure the osteocalcin expression by Saos-2 cells cultured on scaffolds for 7, 14, and 21 days. The results show increased levels of osteocalcin with time for all the tested materials. On day 7, the osteocalcin levels of keratin and keratin/HA showed no significant difference as shown in Figure 12. However, the osteocalcin levels of the keratin/HA group were significantly higher than the keratin group on days 14 and 21.

The sequence of events observed *in vitro* is similar to that *in vivo* where differentiation of cells results in decreased cellular proliferation followed by high levels of ALP expression and osteocalcin secretion which occur at the mineralization phase of bone formation (Turksen and Aubin, 1991; Nuttelman et al., 2005). The process of osteogenic differentiation of cells can be divided into three main phases. During the first phase, there is a rapid cellular proliferation (Feroz and Dias, 2021b). The results of this study are in accordance this but also showed a significantly higher percentage of cell proliferation for keratin scaffolds compared to keratin/HA scaffolds. We postulated that the presence of a higher concentration of hydroxyapatite induced the osteogenic differentiation of cells which resulted in a lower percentage of cell proliferation for keratin/HA scaffolds (Chen et al., 2017). This assumption was later confirmed by the results of ALP analysis which showed significantly higher levels of ALP expression for keratin/HA scaffolds compared to pure keratin scaffolds on days 7, 10, and 14. The second phase of osteogenesis is mainly characterized by the expression of ALP which usually peaked at the first week and therefore, is considered an early marker for cellular differentiation (Chen et al., 2020). The third phase is the deposition of calcium content and the release of some late biochemical markers such as osteocalcin, indicating the later stages of osteogenic differentiation (Neve et al., 2013).

The overall results indicate the osteo-inductive characteristics of the keratin and keratin/HA scaffolds. Furthermore, it shows that both the tested materials can serve as excellent substrates for the differentiation of Saos-2 cells leading to mineralization of the extracellular matrix. This study demonstrates that keratin/HA bi-layered scaffolds possessing osteo-inductive features suitable for the attachment, proliferation, migration, and differentiation of bone cells to potentially enhance the regeneration of new bone.

## 4 Conclusion and future directions

All the optimized factors related to structural designing of the biomimetic scaffolds such as fabrication technique, shape, pore size, and seamless integration of layers influence the final properties of the construct. In current project, we successfully fabricated keratin-based bone grafts having compressive strength value within the range of trabecular part of alveolar bone. Furthermore, the detailed *in vitro* analysis related to morphology, mechanical and biocompatibility of Keratin/HA scaffold showed its potential for various application in bone tissue engineering.

However detailed studies to better investigate the molecular level interaction of keratin-based composite with other natural or synthetic polymers will be necessary to significantly increase their share in commercial market. Furthermore, there is also a dire demand of detailed *in vivo* investigations to better understand the potential application of keratin derived biomaterials for bone tissue engineering at various sites including alveolar bone.

## Data Availability

The original contributions presented in the study are included in the article/Supplementary material, further inquiries can be directed to the corresponding authors.
